# Turkish society’s perception of nursing image during the COVID-19 pandemic

**DOI:** 10.1186/s12912-024-01752-z

**Published:** 2024-02-04

**Authors:** Derya Gündüz Hoşgör, Filiz Coşkun

**Affiliations:** 1 Usak University Medical Faculty-Trainig and Research Hospital, Fevzi Cakmak District Denizli Street No:4, 64100 Central, Usak, Turkey; 2 Program of Operating Room Services, Health Sciences University, Hamidiye Health Services Vocational School, Selimiye, Tıbbiye Street No: 38, 34668 Uskudar, Istanbul, Turkey

**Keywords:** COVID-19, Pandemic, Nursing, Nursing image, Turkish society, Society’s perception

## Abstract

**Background:**

The status of nurses who form the backbone of the health system, and the society’s perspective on nursing has undergone serious transformations during the COVID-19 pandemic. The visibility of nurses, who constantly fought on the front lines in the harsh conditions of the pandemic, increased even more in this period. Thus, this study was aimed at determining Turkish people’s perception of nursing during the COVID-19 pandemic and investigating whether there is a significant relationship between the mean score obtained from the Nursing Image Scale and the descriptive variables.

**Method:**

The sample of this cross-sectional study consisted of 420 Turkish citizens. The “Descriptive Characteristics Form” and “Nursing Image Scale” were used to collect data. In addition to descriptive analysis, the t-test and One-Way ANOVA test were used.

**Results:**

Turkish people’s nursing image during the COVID-19 pandemic was highly positive. Individuals who stated that they had a chronic disease perceived nursing as a professional occupation, which transformed their current nursing image to a more positive image during the COVID-19 pandemic and the mean score they obtained from the Nursing Image Scale was statistically significantly high.

**Conclusion:**

We concluded that Turkish people’s perceptions of nurses changed for the better during the pandemic and their awareness of nursing improved. It is the nurses’ responsibility to improve the nursing image of the society. Therefore, it is critical for nurses to create a modern nurse image picturing them as producers of scientific knowledge and independent leaders.

## Introduction

The COVID-19 pandemic, which has affected the whole world, has affected many sectors and professions. One of these is the healthcare sector and nursing profession. With the pandemic, duties and roles of nurses in the field of health have expanded, which has led to a significant increase both in societies’ and in health professionals’ expectation that nurses should be excellent [1].

Professionalism of nursing has been an important issue in the history of nursing, and a significant progress has been observed in the professionalism of nursing, especially in the last few years after the COVID-19 pandemic emerged. It is known that both the change in the public image of nurses and the social acceptance of nursing as a profession have played a great role in this progress [2].

## Background

Nursing image is closely related to nurses’ identity and roles, their cultural background, reputation, clinical practices, job opportunities, job satisfaction, intention to stay in the profession, quality of care they provide, and thus to the well-being of individuals, families, and communities [3]. One of the long-term challenges faced by nursing since Florence Nightingale has been its image in the public [4].

Since the outbreak of the COVID-19 pandemic, the current image of nurses has changed to brave and tireless healthcare providers who are constantly in the front lines of a fight against a difficult pandemic. The view that nurses are heroes has been reflected in the media and it has strengthened the virtuous image of the nursing profession, largely by changing previous stereotypical perceptions regarding nurses into positive ones [5].

In a systematic review in which society’s perceptions of nursing image was investigated, it was reported that nursing had an important place in public health [6]. On the other hand, it has been revealed that nurses are perceived as subordinates to physicians that provide medical treatments requested by physicians, fulfill physicians’ requests unconditionally, and have long working hours and low wages. It is known that post-pandemic nursing images of societies were different from their pre-pandemic nursing images. For instance, Koç and Sağlam stated that in the pre-pandemic period, a large part of the society perceived nursing as a profession which could not produce evidence-based knowledge and did not require training and expertise [7]. In a study conducted with university students studying in health and non-health fields in Turkey, half of the participants defined the nurse as someone who only injects medication and measures blood pressure [8]. Uysal and Demirdağ conducted a study on Turkish society’s perception of nursing image during the COVID-19 pandemic, and they determined that individuals’ post-pandemic perceptions of nursing were more positive than were their pre-pandemic perceptions. They also determined that in the society, nursing was accepted as a well-known and respected profession with difficult working conditions. However, they also reported that in 71.5% of the people, some traditional stereotypes such as “the nurse is the doctor’s assistant” still prevail [9]. In studies carried out on the issue after the COVID-19 pandemic in Turkish society, health professionals obtained a moderate mean score from the Nursing Image Scale [10, 11]. As in other professions, the opinions, perceptions and expectations of the society are valuable in terms of the development of nursing [12]. Therefore, analyzing and interpreting how nursing is perceived by the society during the development process of nursing is extremely critical in establishing a sustainable image policy. Our literature review demonstrated that the number of studies in which how the COVID-19 pandemic affected perception of nursing and nursing image was investigated was very few and that the existing studies were mostly conducted on nursing students’ perceptions. Therefore, in the present study, it was aimed to investigate Turkish people’s perception of nursing image during the COVID-19 pandemic.

## Methods

### Research design and setting

This study has a cross-sectional quantitative design.

### Participant and sampling

According to Gürbüz and Şahin, using simple random or systematic sampling techniques in cases where the population is spread over a large area is inconvenient; thus, they recommend that the clustering method should be used [13]. Therefore, we selected the sample of the study using the clustering method, which is one of the probability sampling methods. Cluster sampling is performed by selecting one or more clusters after the universe is divided into various clusters [14]. The reason for using the cluster sampling method is that the population is dispersed and the cost of reaching the whole population is rather high [15]. Thus, the data were collected from 198,284 ≥ 18-year-old people living in the provinces with the highest number of COVID-19 cases (Istanbul, Ankara, Izmir, Adana, Samsun and Sanlıurfa) utilizing the case density map included in the latest weekly status report published by the Ministry of Health of the Republic of Turkey using the survey method [16].

We used the sample calculation formula applied in quantitative research to determine the minimum sample size of the study. The formula used is shown below [17]. According to the sample calculation, it was determined that a sample with a minimum of 384 people was sufficient to represent a population of 198,284 people [18]. After their informed consent was obtained, the data obtained from the 420 individuals participating in the study were analyzed.

$$ n=\frac{{z}^{2}*p*q*N}{{d}^{2}*N+{z}^{2}*p*q}$$

N: the number of units in the population.

n: the number of units in the sample.

z: t-statistic at certain degree of freedom and detected error level (1.96).

p: the probability of occurrence of the investigated event (0.5).

q: complement of p (0.5).

d: sampling error (0.05).

### Data collection instruments

The data of this study were collected in April 2021. The e-survey link prepared using Google Forms was sent to people living in these provinces via e-mail, WhatsApp and other social media platforms. The “Descriptive Characteristics Form” and “Nursing Image Scale” were used to collect data.

### Descriptive characteristics form

The form has eight items questioning the following: age, sex, marital status and education level of the participant, whether the participant has a chronic disease, whether the participant has individuals in his or her family / immediate circle who work as a nurse, whether the participant perceives nursing as a profession, and whether the COVID-19 pandemic has changed the participant’s perspective of nursing.

### Nursing image scale

Ozsoy developed the scale as a questionnaire, but did not analyze its psychometric properties [19]. Cinar and Demir made the necessary analysis in order to administer it as a scale [20]. The scale consists of 3 sub-dimensions and 28 items questioning participants’ opinions about the image of the nursing profession. The “General Appearance” sub-dimension has 7 items, the “Communication” sub-dimension has 6 items and the “Professional and Educational Qualifications” sub-dimension has 15 items. The Cronbach’s Alpha (Cα) coefficient was 0.81 for the overall scale, 0.60 for the General Appearance sub-dimension, 0.74 for the Communication sub-dimension and 0.75 for the Professional and Educational Qualifications sub-dimension. In our study, the Cα coefficient was 0.86 for the overall scale, and ranged between 0.62 and 0.87 for the sub-dimensions, which indicates that the scale has a high internal reliability in general.

In the General Appearance sub-dimension, the following are questioned: whether the nurse is well groomed, clean, polite, respectful, cheerful, smiling and attractive, and whether he or she wears a uniform. In the Communication sub-dimension, the following qualifications of nurses are questioned: whether they listen to people, keep secrets, find solutions to patient problems, allow patients to ask questions, and guide patients. In the Professional and Educational Qualifications sub-dimension, the following are questioned: whether nurses should have a university degree, whether they can serve as managers, researchers or academicians, whether their working conditions are heavy, whether nursing is a profession that requires knowledge and skills, whether nurses are respected in the society and whether they play a vital role in the recovery of patients.

Responses given to the items are rated on a three-point Likert type scale ranging from 1 to 3 (1: disagree, 2: somewhat agree, 3: Agree). Items 4 and 6 are reverse scored. The minimum and maximum possible scores to be obtained from the scale are 28 and 84, respectively. However, there are no cut-off points such as low, medium or high in the scale. All the assessments are made based on the mean scores. The higher the score obtained from the overall scale and its sub-dimensions is the more positive the participant’s perception of nursing image is.

### Ethical approval

Before the study was conducted, the approval was obtained from the Uşak University Social and Humanities Scientific Research and Publication Ethics Committee (decision date: March 09, 2021; decision number: 2021-48). The study was conducted in accordance with the Declaration of Helsinki.

### Statistical analysis

The SPSS (IBM Corp. Released 2013. IBM SPSS Statistic for Windows, Version 26.0. Armonk, NY: IBM Corporation) was used in the analysis of the collected data. Within the scope of the study, difference tests were used in addition to descriptive statistics such as percentage, frequency, arithmetic mean, standard deviation, minimum-maximum values. The kurtosis and skewness values ranging between − 1 and + 1 for the overall scale and its sub-dimensions indicate that the data were distributed normally [21]. Therefore, of the difference tests, the t-test and One-Way ANOVA test were used to assess parametric distribution. The Tukey HSD post-hoc test was used to analyze the results which were significant according to the One-Way ANOVA test. In the analysis of the findings obtained, 95% confidence interval and *p* < 0.05 statistical significance level were taken as basis.

## Results

The mean age of the participants was 28.6 ± 10.6 years. Of them, 81.0% were women, 62.1% were single, 79.7% were university graduates, 88.8% did not have any chronic disease, 65.2% had individuals in their family / immediate circle who chose nursing as a profession, 95.2% perceived nursing as a profession, and 46.0% stated that the COVID-19 pandemic positively changed their perspectives of nursing (Table 1).

**Table 1:** Descriptive characteristics of the participants

Descriptive Characteristics		n	%
**Age** ( $\overline x$: 28.6 ± 10.6)	≤ 29 years	245	58.3
	≥ 30 years	175	41.7
**Sex**	Women	340	81.0
	Men	80	19.0
**Marital status**	Married	159	37.9
	Single	261	62.1
**Educational status**	≤ High school	22	5.3
	=University	335	79.7
	≥ Postgraduate	63	15.0
**Do you have a chronic illness?**	Yes	47	11.2
	No	373	88.8
**Is there anyone in your family / immediate circle who has chosen nursing as a profession?**	Yes	274	65.2
	No	146	34.8
**Is Nursing a Profession?**	Yes	400	95.2
	No	20	4.8
**Has the COVID-19 Pandemic Changed Your Perspective on Nursing?**	No	204	48.6
	Yes, positively	193	46.0
	Yes, but negatively	23	5.4
**TOTAL**		**420**	**100.0**

**Abbreviations**: n: Number; %: Percentage; $\overline x$: Mean; ±: Standard Deviation

The mean scores the participants obtained from the overall “Nursing Image Scale” and its “General Appearance”, “Communication” and “Professional and Educational Qualifications” sub-dimensions were 69.09 ± 7.83, 14.47 ± 1.99, 14.87 ± 2.87, and 40.75 ± 4.72 respectively. All the mean scores were higher than was the average score (Table 2).

**Table 2:** Mean scores the participants obtained from the overall nursing image scale and its sub-dimensions

Nursing Image Scale and its Sub-Dimensions	NoI	Min.	Max.	x̄	±	K	S	Cα
**General Appearance**	7	7	21	14.47	1.99	-0.28	-0.35	0.62
**Communication**	6	6	18	14.87	2.87	-0.62	-0.59	0.87
**Professional and Educational Qualifications**	15	15	45	40.75	4.72	0.54	-0.81	0.78
**Nursing Image Scale (Total)**	28	28	84	69.09	7.83	0.41	-0.66	0.86

**Abbreviations**: NoI: Number of the Items; Min: Minimum; Max: Maximum; K: Kurtosis; S: Skewness

In our study, there was no statistically significant difference between the participants in terms of the relationship between the sex variable and the mean scores they obtained from the overall “Nursing Image Scale” and its sub-dimensions (*p* > 0.05). However, there was a statistically significant difference between the participants in terms of the relationship between the age variable and the mean scores they obtained from the “Communication” and “Vocational and Educational Qualifications” sub-dimensions (*p* < 0.05). The significant difference for both sub-dimensions stemmed from the participants aged ≥ 30 years. While the significant difference was in favor of the single participants in terms of “the relationship between the marital status” variable and the mean score for the “Communication” sub-dimension, it was in favor of the participants with a chronic disease in terms of the “relationship between the presence of a chronic disease” variable and the mean scores for the overall “Nursing Image Scale” and its “Communication” sub-dimension (*p* < 0.05). The significant difference was in favor of the participants who had individual(s) in their family/immediate circle who preferred nursing as a profession in terms of the relationship between “the presence of an individual in the family / immediate circle choosing nursing as a profession” variable and the “Communication” sub-dimension (*p* < 0.05) (Table 3).

**Table 3:** Comparisons of the participants’ descriptive characteristics with the nursing image scale and its sub-dimensions

Variable	n	%	NIS	GA	C	PEQ	
**Age**			$\overline x$ ±	$\overline x$ ±	$\overline x$ ±	$\overline x$ ±	
≤ 29 years	245	58.3	2.473 ± 0.283	2.379 ± 0.284	2.372 ± 0.492	2.516 ± 0.303	
≥ 30 years	175	41.7	2.461 ± 0.276	2.327 ± 0.275	2.553 ± 0454	2.591 ± 0.278	
Test Significance			t = 0.425; *p* = 0.671	t = 1.892; *p* = 0.059	**t = 3.885;***p* = 0.000*	**t=-2.595;***p* = 0.010*	
**Sex**							
Women	340	81.0	2.466 ± 0.280	2.356 ± 0.285	2.479 ± 0.477	2.552 ± 0.292	
Men	80	19.0	2.477 ± 0.280	2.361 ± 0.266	2.471 ± 0.485	2.527 ± 0.310	
Test Significance			t=-0.323; *p* = 0.747	t=-0.126; *p* = 0.900	t = 0.144; *p* = 0.885	t = 0.694; *p* = 0.488	
**Marital status**							
Married	159	37.9	2.456 ± 0.286	2.325 ± 0.282	2.387 ± 0.494	2.583 ± 0.295	
Single	261	62.1	2.475 ± 0.276	2.377 ± 0.279	2.533 ± 0.460	2.525 ± 0.294	
Test Significance			t=-0.695; *p* = 0.488	t=-1.820; *p* = 0.070	**t=-3.077;***p* = 0.002*	t = 1.946; *p* = 0.052	
**Do you have a chronic illness?**							
Yes	47	11.2	2.574 ± 0.266	2.422 ± 0.254	2.670 ± 0.360	2.621 ± 0.299	
No	373	88.8	2.454 ± 0.278	2.348 ± 0.283	2.453 ± 0.485	2.537 ± 0.293	
Test Significance			**t = 2.799;***p* = 0.005*	t = 1.695; *p* = 0.091	**t = 2.957;***p* = 0.003*	t = 1.826; *p* = 0.069	
**Is there anyone in your family / immediate circle who has chosen nursing as a profession?**							
Yes	274	65.2	2.474 ± 0.284	2.356 ± 0.290	2.512 ± 0.467	2.549 ± 0.298	
No	146	34.8	2.456 ± 0.272	2.359 ± 0.256	2.413 ± 0.493	2.543 ± 0.291	
Test Significance			t=-0.626; *p* = 0.532	t=-0.104; *p* = 0.917	**t = 2.028;***p* = 0.043*	t = 0.199; *p* = 0.842	
**Is Nursing a Profession?**			$\overline x$ ±	$\overline x$ ±	$\overline x$ ±	$\overline x$ ±	
Yes	400	95.2	2.487 ± 0.262	2.370 ± 0.272	2.503 ± 0.459		2.567 ± 0.278
No	20	4.8	2.073 ± 0.332	2.100 ± 0.344	1.975 ± 0.568		2.159 ± 0.362
Test Significance			**t = 6.801;***p* = 0.000*	**t = 4.277;***p* = 0.000*	**t = 4.957;***p* = 0.000*		**t = 6.295;***p* = 0.000*
**Educational status**							
≤ High school ^a^	22	5.3	2.561 ± 0.282	2.363 ± 0.274	2.359 ± 0.448		2.529 ± 0.296
= University ^b^	335	79.7	2.460 ± 0.285	2.371 ± 0.289	2.485 ± 0.483		2.582 ± 0.321
≥ Postgraduate ^c^	63	15.0	2.473 ± 0.241	2.278 ± 0.225	2.704 ± 0.377		2.631 ± 0.265
Test Significance			F = 1.375; *p* = 0.254	F = 1.903; *p* = 0.072	**F = 4.514;*p*** = 0.012* (c > b > a)		**F = 3.362;*p*** = 0.036* (c > b > a)
**Has the COVID-19 Pandemic Changed Your Perspective on Nursing?**							
No ^a^	204	48.6	2.450 ± 0.291	2.367 ± 0.295	2.432 ± 0.495		2.532 ± 0.308
Yes, positively ^b^	193	46.0	2.504 ± 0.248	2.367 ± 0.251	2.551 ± 0.430		2.578 ± 0.266
Yes, but negatively ^c^	23	5.4	2.310 ± 0.354	2.173 ± 0.330	2.260 ± 0.589		2.415 ± 0.365
Test Significance			**F = 5.776;*p*** = 0.003* (b > c)	**F = 5.272;*p*** = 0.005* (b > c)	**F = 5.744;*p*** = 0.003* (b > c)		**F = 3.625;*p*** = 0.027* (b > c)

**Abbreviations**: * *p* < 0.05; F: One-Way ANOVA testi; t: Independent-samples t test;

**Acronyms**: NIS: Nursing Image Scale; GA: General Appearance; C: Communication; PEQ: Professional and Educational Qualifications

There was a statistically significant difference between the participants in terms of the relationship between the variable “whether nursing is a profession” and the mean scores they obtained from the overall “Nursing Image Scale” and its “General Appearance”, “Communication” and “Professional and Educational Qualifications” sub-dimensions (*p* < 0.05). The significant difference for the scale and all the sub-dimensions stemmed from the participants who considered nursing as a profession. The significant difference was in favor of the participants who had postgraduate education in terms of the relationship between “the education status” variable and their mean scores for the “Communication” and “Vocational and Educational Qualifications” sub-dimensions (*p* < 0.05). The significant difference was in favor of the participants who said that the COVID-19 pandemic changed their perspective of nursing positively in terms of the variable “whether the COVID-19 pandemic changed their perspective of nursing” and their mean scores for the overall “Nursing Image Scale”, and its “General Appearance”, “Communication” and “Vocational and Educational Qualifications” sub-dimensions (*p* < 0.05) (Table 3).

## Discussion

In the present study carried out to investigate the society’s perception of nursing image, 420 Turkish citizens’ perceptions of’ nursing during the COVID-19 pandemic were analyzed. We concluded that almost all of the participants perceived nursing as a profession, and nearly half of them stated that their perspectives regarding nursing profession changed positively due to the COVID-19 pandemic. These results can be interpreted that the public felt the support of nurses more intensely in a challenging atmosphere dominated by a highly contagious and even deadly virus. In addition, these results are promising because they indicate that the image of nursing in Turkish society has gained a positive momentum during the pandemic.

It can be said that the news in the press or the videos on social media about nurses during the pandemic had sometimes positive reflections on the current image of nursing. For instance, during the COVID-19 pandemic in Turkey, Turkish people’s perception of nursing image changed for the better because nurses continued to care for infected patients although their workload was excessively high and they lacked personal protective equipment and medication, among health workers, they were most vulnerable to the disease, they did not go home to avoid infecting their family members/relatives, they were stigmatized by the society due to their likelihood to transmit the COVID-19 and given the ratio of nurses to the general population, the number of nurses was insufficient [22, 23]. It was reported that the rate of Turkish people whose perceptions of nursing changed for the better during the pandemic was 66.2% in Uysal and Demirdağ’s [9] study and 55.3% in Büyükbayram and Aksoy’s study [12]. As in Turkey, in many countries of the world, nurses are known to be at the forefront in the fight against the COVID-19 pandemic, which made the presence and visibility of nurses more obvious [24]. The results of both studies were consistent with those of our study. Similarly, in a study conducted with 80 individuals in Israel, after the COVID-19 pandemic, society’s perceptions of and attitudes towards nursing were more positive than were their perceptions of and attitudes towards other professions [25]. In a study conducted in Italy, Italian people displayed a positive attitude towards nurses and they defined nurses as “heroes” [26]. In addition, it was stated that the mass media led to a positive change in the image for nurses and accordingly, and that some of the participants’ opinions about nurses became positive. Based on this, it can be stated that our study results overlop with Uysal and Demirdağ [9], Büyükbayram and Aksoy [12], Blau et al. [25], and Foà et al. [26].

The results of the present study revealed that the mean scores the participants obtained from the overall Nursing Image Scale and its sub-dimensions regarding their perception of nursing image were high. In a study conducted in Malaysia by Saidun and Akhmetova in 2021, it was revealed that people’s perception of nursing image which was previously negative became positive, and that today, the society perceived nursing as a noble profession [27]. The following statements reported in a qualitative study conducted with Polish nurses by Walowska and Domaradzki in 2023 on their perspectives of nursing after the COVID-19 pandemic were quite noteworthy [28]:

*“For the first time in 25 years, I heard someone appreciated my profession. I hope that the pandemic will eventually change the image of Polish nurses for the better. Even thinking about this makes me happy.”*

*“While I was working in the COVID-19 ward, a 36-year-old man in a shift thanked me for everything. He said that nurses did a great job and they should be praised for it. I spoke to him for a moment and realized that his previous experience was not good, but now he understands what working as nurse is like and appreciates our work.”*

*“People have finally understood that nursing is an independent profession whose members assume responsibility.”*

The analysis of the current findings obtained after the Nursing Image Scale was administered demonstrated that the participants perceived nursing as a profession and their perspectives on nursing turned positive during the pandemic. Of the participants in the present study, those who had a chronic disease, were ≥ 30 years old, were single, had postgraduate education, or had someone in their family/immediate circle who chose nursing as a profession obtained a higher mean score from the Communication subscale. However, of the participants, those who had postgraduate education or were ≥ 30 years old obtained a higher mean score from the Professional and Educational Qualifications sub-dimension.

It is obvious that the results indicating that older people and individuals with chronic diseases are likely to be in need of health care services more than are others and that the pandemic may increase this need are not surprising because these individuals present to hospital more frequently and thus their probability of meeting nurses and receiving care from them will be relatively higher. For example, a patient hospitalized in the COVID-19 wards during the pandemic may have had the opportunity to observe the workload and professional performance of nurses more closely. The presence of nurses who provide uninterrupted service to patients in difficult working conditions where the risk of infection is high may have aroused a feeling of gratitude in the patient and/or patient’s relatives, which may have positively affected their perceptions of nursing. Gratitude is an important issue in Turkish culture, and it reflects the feeling of appreciation for a kind and helpful behavior. With our study findings Yurtsever et al. [29] is similar, while Tarhan et al. [30] is different.

The fact that individuals with postgraduate education have relatively higher knowledge of nursing and nursing education suggests that these results are expected. It is natural for individuals with higher education to have a positive perception of nursing image because they are aware that nurses undergo an undergraduate education, that they can specialize in their fields by gaining master’s or doctorate degree, and that they can become academics. It was concluded that the results of this study are similar to the study results of S. Çelik et al. [31], while the study results of Mat and Baykal [32] are different.

It is not surprising that individuals who think that nursing is a profession, who are familiar with nursing due to having someone who is a nurse in their immediate circle, and who state that their perspective of nursing has turned positive due to the pandemic have a relatively positive image of nursing because it is highly likely that positive perceptions, attitudes and awareness about a profession have positive reflections on the cumulative image of that profession. In their study conducted with 220 nurses in Iran, Varaei et al. determined that the variables “having a nurse family member” and “working in a hospital” had the greatest effect on the formation of nursing image [33]. In their study conducted in Saudi Arabia with 502 individuals, Elmorshedy et al. demonstrated that of the participants, those who were married, had at least a university degree, and had a female nurse family member had a significantly higher level of awareness of the nursing profession [34]. On the other hand, it is obvious that a greater number of empirical studies should be conducted to interpret the results indicating that single people’s perception of nursing image is more positive than is that of married people and that the sex variable does not lead to a significant difference. In this context, Yurtsever et al. [29] study results were found to be similar to this study.

Our review of the literature revealed that results of some studies were consistent with our current results while results of some other studies were not. In a meta-analytic study whose results were consistent with ours, Özkan investigated the effect of sex variable on nursing image and concluded that in Turkey, nurses’ sex did not have a statistically significant effect on people’s perceptions of nursing image [35]. Just like in the present study, in several studies, it has been reported that there is no statistically significant relationship between the nursing image of the society and variables such as age, marital status, sex, education level and income level, which is noteworthy [29, 31, 36].

In their study whose results were different from our study’s results, B. Mat and Baykal’s revealed that socio-demographic and socio-economic variables such as sex, education level, occupation and economic level affected people’s perception of nursing image [32]. In their study conducted with 428 nurses working in a private hospital, Tarhan et al. reported that of the participants, those who were women, those who were younger than 22 years old, those who had less than one year of professional experience, those who worked more than 55 h a week, and those who were willing to choose nursing as a career again had a significantly more positive nursing image [30].

Improving the society’s perception of nursing image is essentially the responsibility of nurses. In the last three years during the COVID-19 pandemic, nurses successfully fulfilled many roles and increased their visibility in the print media and social media, which positively affected the image of nursing in the society and contributed to the professional identity of nursing. As Uysal and Demirdağ stated, such a change can be perceived as a step towards the recognition of nursing as a profession by the society and improvement of its status [9]. On the other hand, it is very important that this step be used effectively by the decision-making mechanisms in health, because at this point, legislators in the health system can contribute to the rise of nursing to an international dimension by developing institutional and legal strategies in order to improve society’s perception of nursing. They can plan awareness-raising activities about the importance of nursing image in society. In addition, studies on the issue are generally handled from the perspective of nursing students. Therefore, increasing the number of studies in which the issue is addressed from the perspective of society may be more beneficial in terms of filling the gaps in the literature.

### Limitations

The fact that the present study was designed to cover only the eight provinces with the highest number of COVID-19 cases prevents the generalization of its results. In addition, the data were collected online; thus, those who volunteered to fill in the questionnaire but could not do it because they did not have internet access did not participate in the study, which constituted the main limitation of the present study.

## Conclusion

The COVID-19 pandemic has greatly raised the society’s awareness of the importance of healthcare professionals, especially of nurses who are considered as the backbone of strong healthcare systems, worldwide. The results of our study demonstrated that there was a positive change in Turkish people’s perspective on nursing due to the pandemic. Although these results are promising, it is a fact that in Turkey, nursing is still perceived as a low-status profession whose members assist the physician. In addition, in Turkey, the health sector is considered as a profession that will last as long as humanity exists by families. It is known that families with low socio-economic status encourage their children especially female children to become nurses because for nurses, the probability of being unemployed is very low. At this very point, it is critical for nurses to create a modern nurse image picturing them as producers of scientific knowledge and independent leaders.

## Data Availability

No datasets were generated or analysed during the current study.
